# Vacuolar ATPase depletion contributes to dysregulation of endocytosis in bloodstream forms of *Trypanosoma brucei*

**DOI:** 10.1186/s13071-020-04068-4

**Published:** 2020-04-25

**Authors:** Zhi-Shen Xu, Feng-Jun Li, Geoff Hide, Zhao-Rong Lun, De-Hua Lai

**Affiliations:** 1 Center for Parasitic Organisms, State Key Laboratory of Biocontrol, School of Life Sciences, and Key Laboratory of Tropical Disease Control (Sun Yat-Sen University), Ministry of Education, Sun Yat-Sen University, Guangzhou, 510275 The People’s Republic of China; 2 Department of Biological Sciences, National University of Singapore, Singapore, 11754 Singapore; 3 Biomedical Research Centre and Ecosystems and Environment Research Centre, School of Science, Engineering and Environment, University of Salford, Salford, M5 4WT UK

**Keywords:** *Trypanosoma brucei*, Vacuolar ATPase, Apolipoprotein L1, Endocytosis, Trypanolysis

## Abstract

**Background:**

Vacuolar H^+^-ATPase (V-ATPase) is a highly conserved protein complex which hydrolyzes ATP and pumps protons to acidify vacuolar vesicles. Beyond its role in pH maintenance, the involvement of V-ATPase in endocytosis is well documented in mammals and plants but is less clear in *Trypanosoma brucei*.

**Methods:**

In this study, the subcellular localization of V-ATPase subunit B (TbVAB) of *T. brucei* was assessed *via in situ* N-terminal YFP-tagging and immunofluorescence assays. Transgenic bloodstream forms (BSF) of *T. brucei* were generated which comprised either a V-ATPase subunit B (*TbVAB*) conditional knockout or a V-ATPase subunit A (*TbVAA*) knockdown. Acridine orange and BCECF-AM were employed to assess the roles of V-ATPase in the pH regulation of BSF *T. brucei*. The endocytic activities of three markers were also characterized by flow cytometry analyses. Furthermore, trypanosomes were counted from trypanolysis treatment groups (either containing 1% or 5% NHS) and endocytosed trypanosome lytic factor (TLF) was also analyzed by an immunoblotting assay.

**Results:**

TbVAB was found to localize to acidocalcisomes, lysosomes and probably also to endosomes of BSF of *T. brucei* and was demonstrated to be essential for cell growth. *TbVAB* depletion neutralized acidic organelles at 24 hours post-tetracycline depletion (hpd), meanwhile the steady state intracellular pH increased from 7.016 ± 0.013 to 7.422 ± 0.058. Trypanosomes with *TbVAB* depletion at 24 hpd were found to take up more transferrin (2.068 ± 0.277 fold) but less tomato lectin (49.31 ± 22.57%) by endocytosis, while no significant change was detected in dextran uptake. Similar endocytic dysregulated phenotypes were also observed in *TbVAA* knockdown cells. In addition, *TbVAB* depleted trypanosomes showed a low uptake of TLF and exhibited less sensitive to lysis in both 1% and 5% NHS treatments.

**Conclusions:**

TbVAB is a key component of V-ATPase and was found to play a key function in endocytosis as well as exhibiting different effects in a receptor/cargo dependent manner in BSF of *T. brucei*. Besides vacuolar alkalinization, the dysregulation of endocytosis in *TbVAB* depleted *T. brucei* is considered to contribute to the reduced sensitivity to lysis by normal human serum.
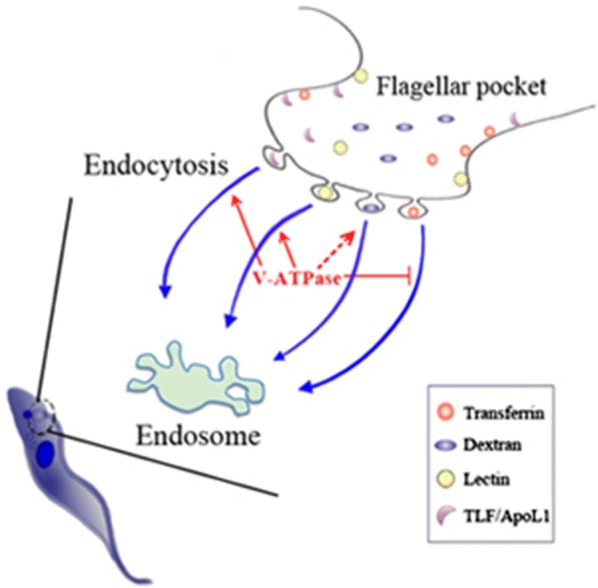

## Background

*Trypanosoma brucei*, a unicellular haemoflagellate, the causative agent of sleeping sickness or human African trypanosomiasis, is threatening approximately 70 million people living in sub-Saharan Africa [1]. *Trypanosoma brucei* evades the mammalian immune system using antigenic variation in the surface coat and relying on a very efficient endocytic system that is capable of recycling the entire surface protein coat within 12 minutes [2]. It also utilizes the serum-resistance associated (SRA) protein in *T. b. rhodesiense* [3] or glycoprotein TgsGP in *T. b. gambiense* [4] to resist trypanolysis mediated by APOL1 in normal human serum (NHS). Recently, genomic-scale RNA interference screening revealed a link between NHS/APOL1-mediated trypanolysis [5, 6] and drug toxicity [7] to vacuolar H^+^-ATPase (V-ATPase) in *T. brucei*, indicating the potential physiological functions of V-ATPase in challenging environments.

V-ATPase is a conserved and crucial protein complex found in endosomal compartments in all eukaryotes [8, 9]. This large complex contains many subunits, forming a rotary cytoplasmic V_1_ moiety (V_1_-ATPase, subunits A_3_B_3_CDE_3_FG_3_H) and a transmembrane Vo moiety (Vo proton channel, subunits ac_8_c′c″de). The concerted action of the V_1_ and Vo moiety couples ATP hydrolysis with the transport of protons across membranes and defines the fundamental function of V-ATPase in cellular compartment acidification. The V-ATPase generates an electrochemical proton gradient as a driving force for numerous secondary transport activities across membranes [10]. Furthermore, V-ATPase can be coupled with other transporters, e.g. the Na^+^/H^+^ antiporter, and contributes to intracellular pH homeostasis in mammalian cells [11]. Previous studies also revealed multi-physiological functions of V-ATPase in response to environmental stresses such as nutrient availability, salinity and pH, *via* an assembly/disassembly mechanism [12–14]. The requirement for V-ATPase activity in secretory and endocytic trafficking was also observed in mammals and plants [15–18], providing important evidence for an extensive function of V-ATPase activity. Although V-ATPase has been well studied in various model organisms, its roles and mechanisms of action in early-branched single cell protozoans is less clear.

Bioinformatic analyses have identified all orthologues of the V-ATPase subunits in *T. brucei* [5] and pioneering studies indicated some important and distinctive roles of the V-ATPase in *T. brucei* [7]. Subunits α (Tb427.05.1300) and d (Tb927.5.550) of V-ATPase in the procyclic form (PCF) found in the insect vector of *T. brucei* was found localized to the lysosomes, Golgi complex and acidocalcisomes [19], indicating localization-dependent distinct roles of V-ATPase. RNAi knockdown of subunit A (*TbVAA*, Tb427.04.1080) and α blocked the acidification of lysosomes and acidocalcisomes in the PCF of *T. brucei* [20]. Treatment of the cells with bafilomycin A_1_, a V-ATPase inhibitor binding to subunit c [21], markedly acidified the steady-state pH_i_ of PCF of *T. brucei* and decreased the pH_i_ recovery rate [22]. The bafilomycin A_1_ could further disturb Ca^2+^ homeostasis in the PCF of *T. brucei via* a Ca^2+^/H^+^ antiporter in a pH gradient-dependent manner [23]. However, for the medically important human infective bloodstream form (BSF) of *T. brucei*, only a few studies have been conducted. Important questions remain unanswered such as whether V-ATPase has a similar localization in the BSF as in the PCF of *T. brucei* and whether V-ATPase has similar roles in endocytic trafficking in BSF of *T. brucei* as in other eukaryotes. Several V-ATPase subunits have been revealed to be crucial for trypanolysis by NHS/recombinant APOL1 [5, 6] and drug toxicity [7], and have been suggested to attribute to V-ATPase’s role in vacuolar acidification [5–7]. However, studies of the roles of trypanosomal V-ATPase activity in endocytosis are preliminary [5] although it has been consistently shown to make a contribution to pH regulation [5, 22].

To better understand the functions of V-ATPase in the BSF of *T. brucei*, we characterized the localization of the V-ATPase subunit B (TbVAB) and investigated its function by using conditional knockout mutants (cKO) or RNAi knockdown (KD) methods. Our results reveal its multiple localizations and crucial roles affecting endocytosis in the BSF of *T. brucei* in a receptor/cargo dependent manner. Our findings also indicate coordination of lysosomal function with upstream endocytic activity in trypanosomes, in which V-ATPase defects may lead to a reduced sensitivity to TLF-mediated trypanolysis.

## Methods

### Cell culture and transfection

*Trypanosoma brucei brucei* cell lines Lister 427, SmOx B427 [24], SmOx PAntat1.1 and derivatives were grown in HMI-9 (for bloodstream forms) or SDM-79 (for procyclic forms) media supplemented with 10% fetal calf serum  (ExCell Bio, Shanghai, China). Transfection was conducted using an Amaxa Nucleofector (Lonza, Cologne, Germany) with human T-cell Nucleofector solution [25] and transfectants were selected with blasticidin, hygromycin, neomycin, phleomycin or puromycin [26]. Cell proliferation was monitored by counting with a hemocytometer every 12 or 24 h.

### Plasmid construction

Gene-specific RNAi fragments (~400 bp) of *TbVAA* (Tb427.04.1080) and *TbVAB* (Tb927.11.11690) were selected with RNAit (https://dag.compbio.dundee.ac.uk/RNAit/), amplified by PCR using primers shown in Additional file 1: Table S1 and cloned into p2T7^Ti^-177 vector [27] using the restriction enzymes indicated. Gene knockout plasmids were generated by insertion of about 500 bp of *TbVAB* 5’ and 3’ UTR into the plew90/plew13 backbone, respectively [28]. To ectopically express the N-terminal TY-tagged *TbVAB*, the full-length ORF was amplified with Q5 HF DNA polymerase (NEB, Ipswich, USA), digested with *Spe*I and *Bcl*I, and then cloned into the pDex577 plasmid [29, 30]. For inducible expression of C-terminal HA-tagged Rab11 [30], the ORF was amplified (a HA-tag was introduced in the reverse primer, Additional file 1: Table S1) and cloned into the pDex577 vector. Linearized pDex577 plasmids were transfected and integrated into the trypanosomes 177 bp telomere repeats through homologous recombination [27]. To construct the N-terminal YFP-tagged *TbVAB*, the 5’ UTR and 5’ CDS fragments were amplified and cloned into *Spe*I and *Bam*HI digested pEnT6B-Y [30].

### Recombinant *TbVAB* expression and antiserum preparation

To express the N-terminal His6-tagged protein, the full length of *TbVAB* ORF was cloned into pET-32 α (+), using primers shown in Additional file 1: Table S1. The resultant vectors were transformed into *E. coli* BL21 (DE3) cells (Transgen, Beijing, China) and protein expression was induced with 0.6 mM isopropyl-β-D-1-thiogalactopyranoside (IPTG) at 37 °C for 5 h. Cells were centrifuged at 10,000× *g* for 5 min and inclusion body proteins were extracted using ultra-sonication. The recombinant proteins were purified using Ni-NTA resin (Qiagen, Valencia, USA) following the manufacturer’s protocol. Rabbits were immunized with purified proteins according to standard procedures. The antisera were stored at -80 °C until used.

### Immunoblotting

Proteins extracted from 2.5 × 10^6^ cells were separated by SDS/PAGE gel and transferred onto NC membrane (PALL, Ann Arbor, USA). The immunoblotting against specific proteins were performed using rabbit anti-TbVAB (1:2000), rabbit anti-APOL1 (1:500, Proteintech Group, Chicago, USA) and mouse anti-PFR (L8C4, 1:500) [31] after blocking in TTBS with 5% milk. The blots were incubated with horseradish peroxidase conjugated anti-mouse or anti-rabbit IgG (H+L) antibody (1:2000, Invitrogen, Carlsbad, USA) and visualized using the ECL substrate (Thermo Fisher Scientific, Rockford, USA). The signal intensity was quantified using Image J.

### Vacuolar and cellular pH measurement

After 30 min incubation at 37 °C with 6 μM acridine orange (AO) (Sigma, St. Louis, USA), and washing with PSG, vacuolar pH of trypanosomes was monitored by fluorescence microscopy using the 590–650 nm filter channel, while DNA contents were also monitored with the 505–550 nm filter channel. The intracellular pH (pH_i_) of trypanosomes was monitored using the cell-permeant, dual-excitation ratiometric pH indicator BCECF-AM (2’, 7’-bis (2-carboxyethyl)-5, 6-carboxyfluorescein acetoxymethyl ester) (Beyotime, Shanghai, China) following the published protocol [22]. Briefly, cells were harvested and washed twice in buffer A (116 mM NaCl, 5.4 mM KCl, 0.8 mM MgSO_4_, 5.5 mM D-glucose and 50 mM Hepes, pH 7.4) at 4 °C and resuspended in loading buffer (135 mM NaCl, 5 mM KCl, 1 mM MgSO_4_, 1 mM CaCl_2_, 15 mM sucrose, 10 mM Hepes at pH 7.4 and 0.1 μM BCECF-AM) to a final density of 1 × 10^8^ cells/ml. After incubation on ice for 30 min, all samples were washed twice with pre-chilled separation buffer (40 mM NaCl, 57 mM Na_2_HPO_4_, 3 mM NaH_2_PO_4_, 10 mM D-glucose at pH 7.4) and resuspended in the same buffer to a final density of 2 × 10^7^ cells/ml. A total of 200 μl cell suspension was transferred into a black 96-well microplate and the fluorescence was monitored at 37 °C using a thermostatically controlled SpectraMax i3x microplate fluorometer (Molecular Devices, Sunnyvale, USA). pHi values in triplicate wells were obtained from three technical replicates and two biological replicates. A standard curve was obtained using cells suspended in loading buffer at pH of 6.2, 6.6, 7.0, 7.4, 7.8 or 8.2 with 0.1 μM BCECF-AM and 2 μM Nigericin. The intracellular pH was calculated based on the standard curve.

### Immunofluorescence microscopy

Cells were settled onto poly-L-lysine treated slides, fixed in 4% formaldehyde and permeabilized with 0.3% Triton X-100. After blocking with 3% BSA in PBS, cells were stained with Rabbit anti-HA polyclonal (1:100, Beyotime, Shanghai, China) and rabbit anti-VP1 polyclonal (1:300) [32] primary antibodies to visualize HA-tagged protein and acidocalcisomes, respectively. As a secondary antibody, TRITC conjugated goat anti-rabbit IgG (H+L) (Invitrogen, Carlsbad, USA) was used. The mitochondrion in *T. brucei* was monitored by microscopy after incubation with 50 nM Mito-Tracker Red CMXRos (Life Technologies, Carlsbad, USA) in HMI-9 for 20 min at 37 °C, followed by washing with PBS. The cells were fixed on slides in 4% formaldehyde and washed in PBS with 0.1 M glycine. DNA contents were stained with DAKO fluorescent mounting medium containing 50 ng/ml DAPI. Images were documented using a Zeiss Axio Imager A2m epifluorescence microscope and a Nikon N-SIM microscope.

### Trypanolysis assay

Wild-type (WT) and *TbVAB* cKO cells were pelleted and resuspended in tetracycline-free HMI-9 medium supplemented with 10% (v/v) FCS and incubated for 8 h at 37 °C. Then cells were diluted to 2 × 10^5^ cells/ml in HMI-9 medium and aliquoted into 48-well plates alongside various concentrations (1% or 5%) of normal human serum (donated by male volunteers in our laboratory). Cells were incubated in triplicate wells at 37 °C with 5% CO_2_ and counted with a hemocytometer every 8 h. Experiments were done with three biological replicates (*n* = 3).

### Endocytosis assays using fluorescence microscopy and flow cytometry

BSF cells in exponential phase were harvested by centrifugation at 1000× g for 10 min at 4 °C, washed in cold PSG and then resuspended to 5 × 10^6^/ml in cold PSG with either 10 μg/ml DyLight 488-tomato lectin (Vector Laboratories, Burlingame, USA) or DyLight 594-tomato lectin (Vector Laboratories, Burlingame, USA), 5 mg/ml 10,000 MW dextran Alexa Fluor 488 (Life Technologies, Carlsbad, USA) or 50 μg/ml transferrin Alexa Fluo 488 (Life Technologies, Carlsbad, USA) [33]. After incubation at 37 °C for series time points, the endocytosis was terminated on ice and half of the cells were pelleted and resuspended to 2.5 × 10^6^/ml in cold PSG with 2% paraformaldehyde and then analyzed by flow cytometry (30,000 counts). The other half of the cells were mounted on slides and monitored by fluorescence microscopy.

### Statistical analysis

Image J software [34] was used to measure the quantitative relation of proteins in western blot assays by gray scanning. Prism® 5.0 software (GraphPad Software Inc) was used to graphically represent data and perform statistical analysis using an unpaired *t*-test. *P*-values of less than 0.05 were considered significant. Data are presented as the mean ± standard deviation (SD).

## Results

### TbVAB is localized in multiple organelles of bloodstream form trypanosomes and essential for cell growth

**Fig. 1:**
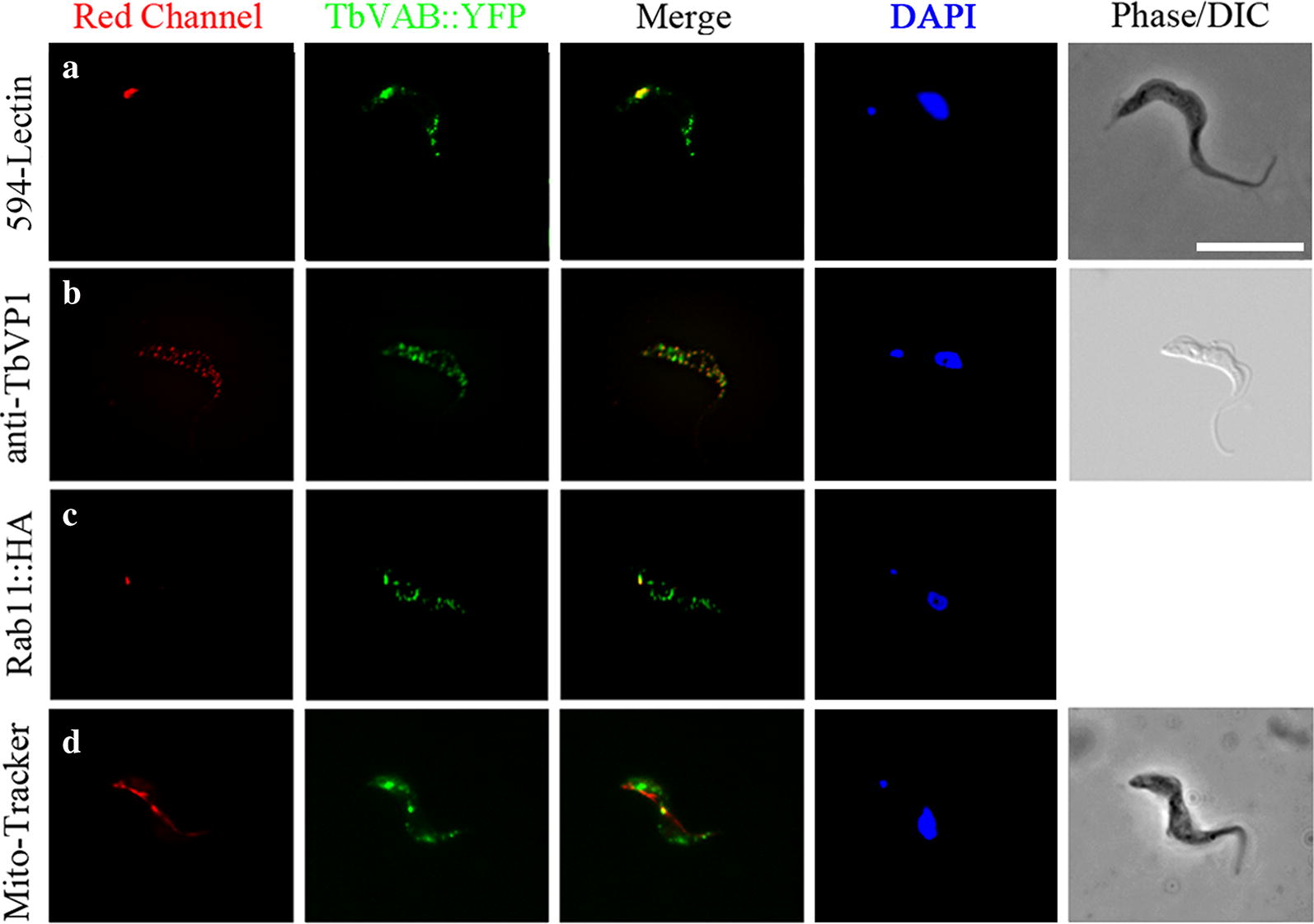
TbVAB localizes to acidocalcisomes, lysosomes and endosomal vesicles in the bloodstream forms of *Trypanosoma brucei*. The endogenous TbVAB::YFP partially co-localized with endocytosed DyLight 594-lectin (**a**), the acidocalcisomes marker TbVP1 (**b**) and the recycling endosomal marker Rab11 (**c**) in BSF of *T. brucei* as shown by immunofluorescence microscopy. No colocalization with Mito-Tracker was observed (**d**). Nuclei and kinetoplasts were stained with 50 ng/ml DAPI. *Scale-bar*: 10 μm

To investigate the localization of vacuolar ATPase in the BSF of *T. brucei*, a cell line expressing YFP-tagged vacuolar ATPase subunit B *in situ* was generated (TbVAB::YFP). Immunoblotting using rabbit polyclonal antibodies against TbVAB showed that both the YFP-tagged *TbVAB* gene and the wild-type *TbVAB* gene were expressed (Additional file 2: Figure S1). We incubated YFP-tagged trypanosomes with 10 μg/ml tomato lectin, at 37 °C for 30 min, which are efficiently taken up and delivered to the lysosome. Fluorescence microscopy revealed multiple intracellular localizations, a few large spots adjacent to the flagellar pocket colocalized with the endocytosed 594-Lectin (Fig. 1a) while punctuated spots diffused through the cytosol colocalized with the acidocalcisomal marker TbVP1 (vacuolar proton pyrophosphatase) (Fig. 1b). Some TbVAB-positive punctuated spots were colocalized with HA-tagged Rab11, a recycling endosomal marker (Fig. 1c). Nevertheless, TbVAB was not found in the mitochondrion as defined by the Mito-Tracker dye (Fig. 1d).

**Fig. 2:**
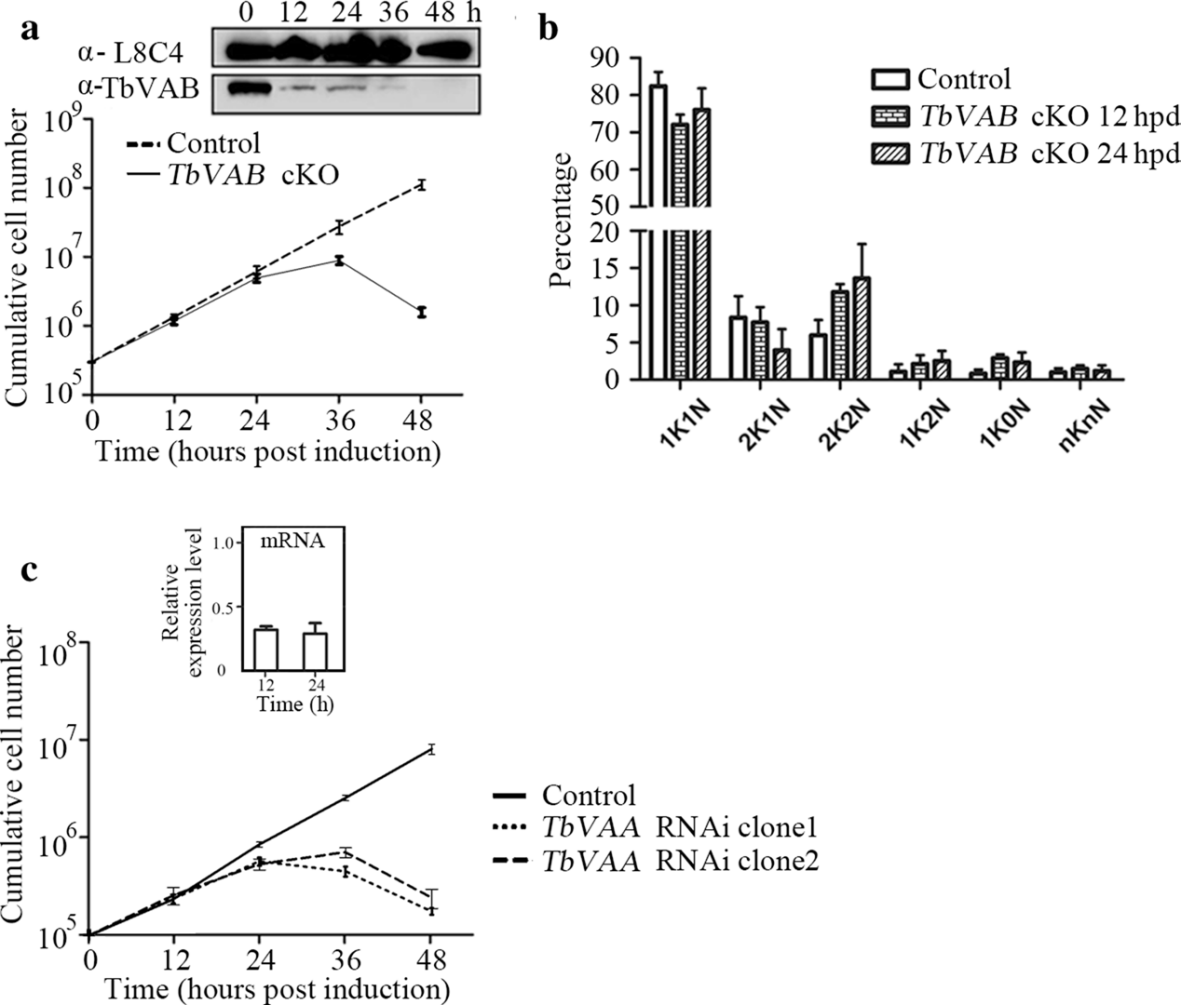
Growth curves of bloodstream forms of *Trypanosoma brucei* after depletion of *TbVAB* and *TbVAA*. **a** Cumulative growth curves of the inducible conditional *TbVAB* knockout in *T. brucei* bloodstream SmOx B427 cells. The ectopically *TbVAB* modified cells were grown in HMI-9 (10% FCS) with 1μg/ml tetracycline and used as a control. *TbVAB* expression was detected by Western blot analysis with the internal loading control being monoclonal antibody L8C4 which recognizes the paraflagellar rod. **b** Analysis of kinetoplast (K) and nucleus (N) numbers in the bloodstream form *T. brucei* after knockout of *TbVAB*. Kinetoplasts and nuclei of 510 cells in each group were counted after stained with DAPI. **c** Cumulative growth curve of the inducible knockdown of *TbVAA* in *T. brucei* bloodstream forms. Non-induced trypanosomes were used as control. The extent of TbVAA mRNA knockdown was assayed by qRT-PCR at indicated time points. The TbVAA mRNA expression from biological triplicates was shown with reference to the GAPDH gene

To understand the function of *TbVAB*, a conditional knockout BSF cell line was generated by replacing both *TbVAB* alleles in a tetracycline inducible *TbVAB* expression cell line. The correct gene integrations of both *TbVAB* alleles were confirmed by PCR (Additional file 3: Figure S2) and the disruption of *TbVAB* expression after tetracycline withdrawal was confirmed by immunoblotting using the rabbit polyclonal antibodies against TbVAB (Fig. 2a). *TbVAB* cKO BSF parasites exhibited severe growth defects at 36 h post-tetracycline depletion, but almost no growth defects before 24 hpd (Fig. 2a). When looking at the cell cycle stages, little was altered during the 12 to 24 hpd phase (Fig. 2b), therefore the following experiments were conducted in that same time window. RNAi knockdown of the V-ATPase subunit A (*TbVAA*) in the BSF of *T. brucei* clones led to significant growth defects (Fig. 2c). These data, taken together with similar growth defects caused by knockdown of other V-ATPase subunits in previous studies [5–7], revealed that the integrity of the whole V-ATPase complex is essential for the BSF of *T. brucei*.

### V-ATPase depletion neutralized the acidic organelles and disturbed the intracellular pH homeostasis in BSF trypanosomes

**Fig. 3:**
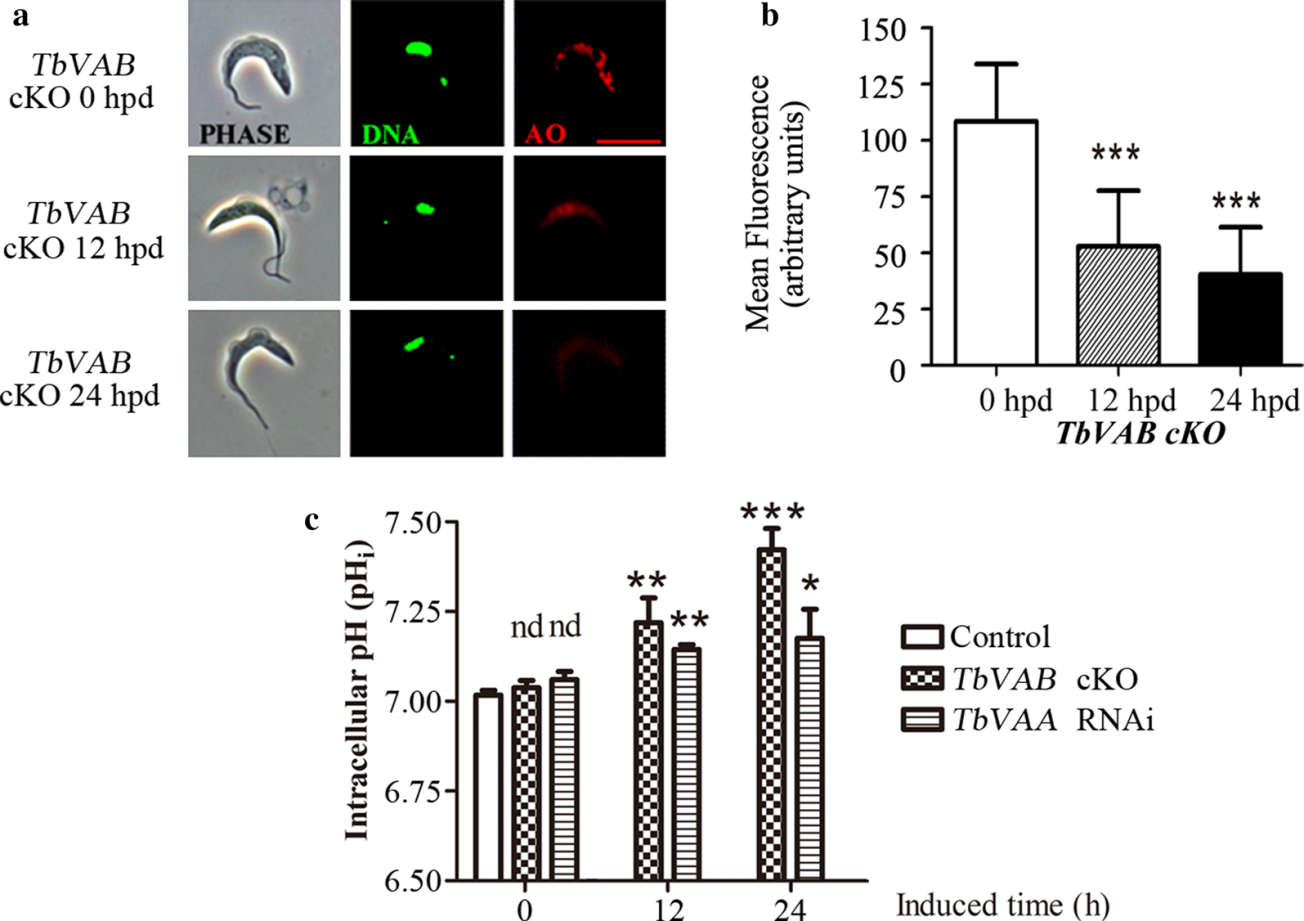
*TbVAA* and *TbVAB* depletion affects vacuolar and cellular pH in bloodstream form *Trypanosoma brucei*. **a** The *TbVAB* cKO BSF trypanosomes were stained with 6 μM acridine orange before and after 12h or 24h with tetracycline withdrawal and the signals were measured by fluorescence microscopy using the 590–650 nm filter channel. The DNA was monitored with the 505–550 nm filter channel. *Scale-bar*: 10 μm. **b** Quantitation of mean AO fluorescent intensities in cKO controls (33 cells), 12-hpd (76 cells) and 24-hpd (79 cells) *TbVAB* cKO cells. The unpaired *t*-test indicated significant differences (***P < 0.001) between control and *TbVAB* cKO cells. Data are representative of three biological replicates. **c** The pH_i_ of *TbVAB* cKO and *TbVAA* knockdown, non-induced control and parental BSF cells were stained with BCECF-AM and measured with microplate fluorometer in triplicate wells from three technical replicates and two biological replicates. Error bars indicate the SD; *P < 0.05, **P < 0.01, ***P < 0.001, nd, no change detected

V-ATPase has well known activity in maintaining the acidic environment of intracellular vacuoles, such as lysosomes and also acidocalcisomes, a lysosome-related organelle, in *T. brucei* [5, 20]. To confirm the specificity of depletion of *TbVAB*, the acidity of these intracellular organelles of *TbVAB* cKO and control cells were monitored with Acridine Orange staining. In comparison with the non-induced control cells, AO red fluorescence intensity was significantly depleted in *TbVAB* cKO cells at 12 hpd (*t*_(35)_ = 6.202, *P* < 0.001) and 24 hpd (*t*_(35)_ = 8.465, *P* < 0.001) (Fig. 3a, b).

The steady state intracellular pH (pH_i_) was monitored using the cell permeable pH indicator BCECF-AM. The *TbVAB* cKO cells at 0 hpd showed a similar level of pH_i_ to the parental cells (7.016 ± 0.013 *versus* 7.038 ± 0.019, *t*_(4)_ = 1.580, *P* = 0.189) (Fig. 3c), whereas at 12 hpd and 24 hpd, the pH_i_ was increased to 7.219 ± 0.068 (*t*_(4)_ = 5.017, *P* = 0.007) and 7.422 ± 0.058 (*t*_(4)_ = 11.560, *P* < 0.001), respectively (Fig. 3c). Similar phenotypes were found in *TbVAA* knockdown cells (Fig. 3c). Taken together, these data suggested that the depletion of *TbVAB* or *TbVAA* resulted in the disruption of intracellular pH homeostasis in the BSF of *T. brucei*.

### Depletion of V-ATPase subunits altered endocytosis in BSF trypanosomes and reduced the sensitivity to NHS-mediated trypanolysis

**Fig. 4:**
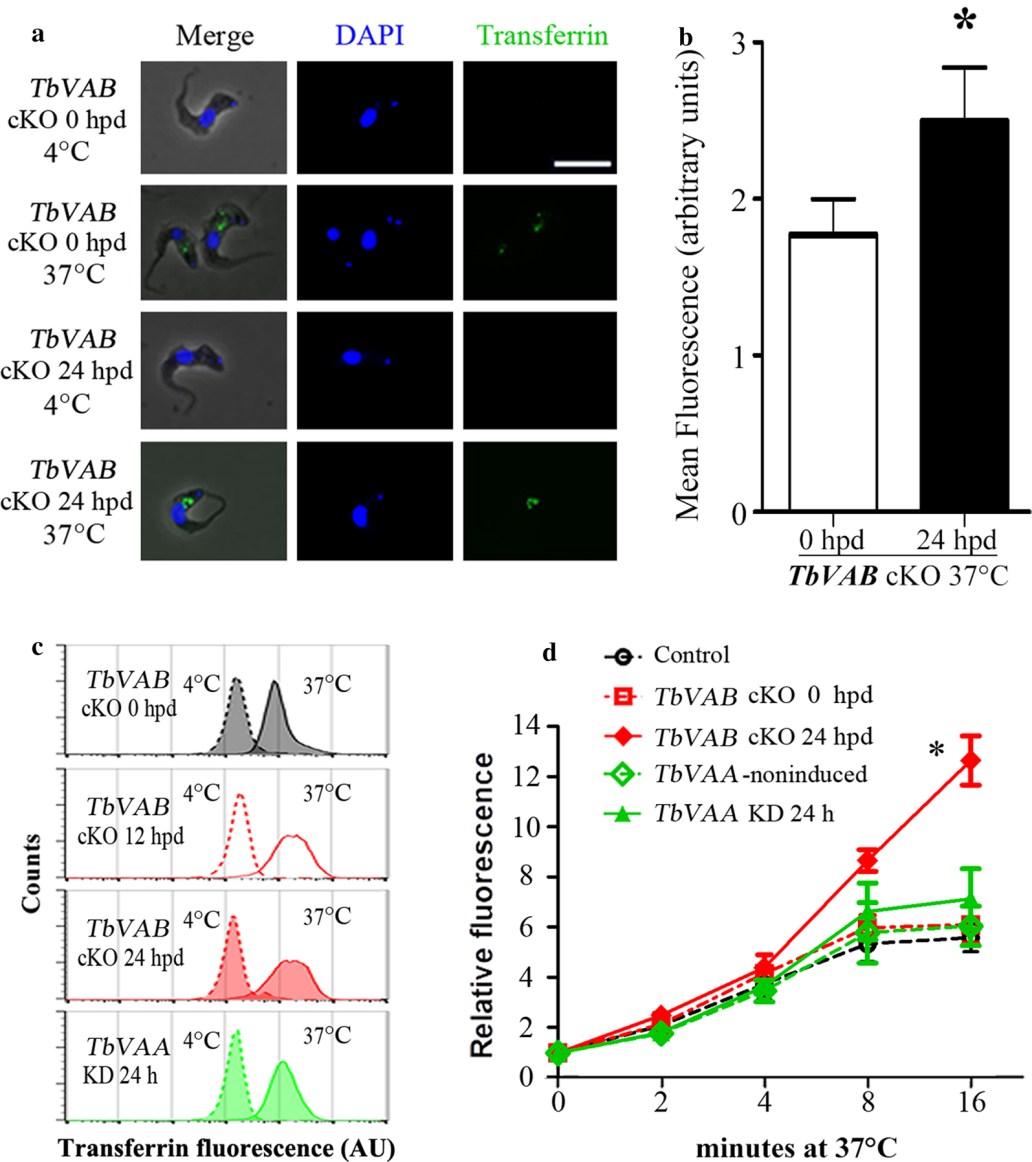
Effect of V-ATPase depletion on the uptake of transferrin in the bloodstream forms of *Trypanosoma brucei*. **a** Trypanosomes were allowed to endocytose transferrin Alexa Fluo 488, at the indicated temperatures for 16 min, and were fixed and stained with DAPI. *TbVAB* depletion in BSF *T. brucei* was induced by withdrawing tetracycline for 24 h before the transferrin incubation. *Scale-bar*: 10 μm. **b** Control and *TbVAB* cKO 24 hpd cells were incubated with transferrin Alexa Fluo 488 at 37 °C for 16 min to allow transferrin uptake. Values represent the mean fluorescence for 72 cells ± SD from three technical replicates (*n* = 1). The unpaired *t*-test indicated a significant difference (*P < 0.05) between control and *TbVAB* cKO 24 hpd cells. **c** Internalization of transferrin increased after the induction of *TbVAB* knockout. Cells were incubated with transferrin Alexa Fluo 488 at 4 °C and then transferred to 37 °C for 16 min to activate endocytosis. Fluorescent intensities distributions of 30,000 cells were analyzed and shown in the flow cytometry diagrams. AU, arbitrary units. **d** Flow cytometry analysis of transferrin uptake after *TbVAB* knockout and *TbVAA* knockdown. Cells were incubated with transferrin Alexa Fluo 488 at 4 °C and subsequently transferred to 37 °C at the times indicated. Mean fluorescence intensity values of 30,000 cells were normalized against the 4 °C incubated cells. Unpaired *t*-test analysis indicated a significant difference (**P < 0.01) between *TbVAB* cKO 0 hpd and 24 hpd cells

Our findings that the V-ATPase subunit TbVAB is localized to lysosomes and probably endosomes (Fig. 1) imply a possible relationship between V-ATPase and endocytic activity. To test this, fluorescence imaging and flow cytometry assays were performed using different surrogate markers: transferrin, tomato lectin and dextran. At 4 °C, the *TbVAB* cKO cells hardly bound transferrin in the flagellar pocket and were similar to the non-induced cells (Fig. 4a). When incubated with transferrin for 16 min at 37 °C, the internalization (endocytosis) of transferrin could be clearly seen in *TbVAB* cKO cells at 0 hpd and were even uplifted at 24 hpd (Fig. 4a, b). Flow cytometry assays were employed to quantify the time course of transferrin accumulation. Compared with the initial status at 0 hpd, the *TbVAB* cKO 24 hpd cells took up significantly more transferrin by endocytosis (2.068 ± 0.277 fold at 16 min, *t*_(4)_ = 5.310, *P* = 0.006) after the 37 °C incubation (Fig. 4c, d). However, no statistically significant difference in transferrin uptake was found in *TbVAA* KD cells (Fig. 4c, d).

**Fig. 5:**
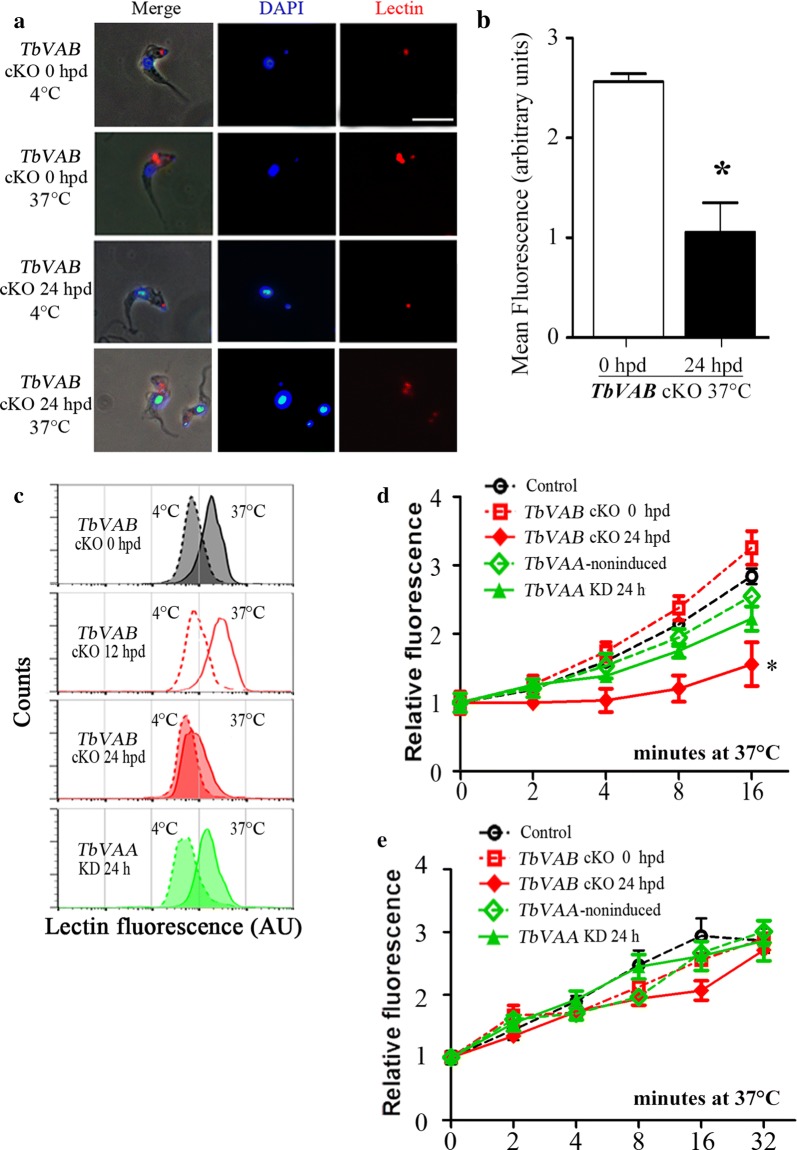
Effects of V-ATPase depletion on the uptake of lectin and dextran in BSF *Trypanosoma brucei*. **a** The BSF trypanosomes were allowed to endocytose DyLight 594-tomato lectin, at the indicated temperatures for 30 min, and were fixed and stained with DAPI. *TbVAB* depletion in BSF *T. brucei* was induced by withdrawing tetracycline for 24 h prior to the tomato lectin incubation. *Scale-bar*: 10 μm. **b** Control and *TbVAB* cKO 24 hpd cells were incubated with DyLight 594-tomato lectin at 37 °C for 30 min to allow lectin uptake. Values represent the mean fluorescence for 33 cells ± SD. The unpaired *t*-test indicated a significant difference (*P < 0.05) between control and *TbVAB* cKO 24 hpd cells. **c** Internalization of tomato lectin decreased after the induction of *TbVAB* knockout (cKO) and *TbVAA* knockdown (KD). Cells were incubated with DyLight 488-tomato lectin at 4 °C and then transferred to 37 °C for 16 min to activate endocytosis. Fluorescent intensities distributions of 30,000 cells were analyzed. AU, arbitrary units. **d**, **e** Flow cytometry analysis of tomato lectin and dextran uptake after *TbVAB* knockout and *TbVAA* knockdown. Cells were incubated with DyLight 488-tomato lectin (**d**) or dextran Alexa Fluor 488 (**e**) at 4 °C and subsequently transferred to 37 °C for the time indicated. Mean fluorescence intensity values of 30,000 cells were normalized against the 4 °C incubated cells. The unpaired *t*-test analysis of lectin uptake indicated a significant difference (*P < 0.05) between *TbVAB* cKO 0 hpd and 24 hpd cells

At 4 °C, the *TbVAB* cKO 24 hpd cells bound tomato lectin mainly in the flagellar pocket and this was similar to the cells at 0 hpd (Fig. 5a). Intriguingly, after incubation at 37 °C for various times, the internalization of tomato lectin was disrupted in the *TbVAB* cKO cells (24 hpd *vs* 0 hpd cells, dropped to 52.67 ± 21.29% at 8 min, *t*_(4)_ = 4.514, *P* = 0.011, or 49.31 ± 22.57% at 16 min, *t*_(4)_ = 4.225, *P* = 0.013; Fig. 5b-d). However, it was only slightly inhibited in the *TbVAA* KD cells (24 h induced *vs* non-induced cells, dropped to 90.05 ± 8.86% at 8 min, *t*_(4)_ = 1.222, *P* = 0.289, or 87.24 ± 11.93% at 16 min, *t*_(4)_ = 1.811, *P* = 0.144, respectively) after the 16 min incubation (Fig. 5c, d). Neither mislocalization of lectin nor transferrin was observed, indicating no major defects in the endomembrane system morphology. To exclude the possibility that the internalization of tomato lectin or transferrin was caused by receptor-independent fluid-phase pinocytosis, the uptake of Dextran Alexa Fluor 488 was measured in control or V-ATPase depleted cells. As shown in Fig. 5e, no significant change in relative fluorescence was detected by measurements of dextran uptake. Taken together, the above results indicated that V-ATPase contributes to the endocytosis process in BSF trypanosomes in a receptor/cargo-dependent manner.

**Fig. 6:**
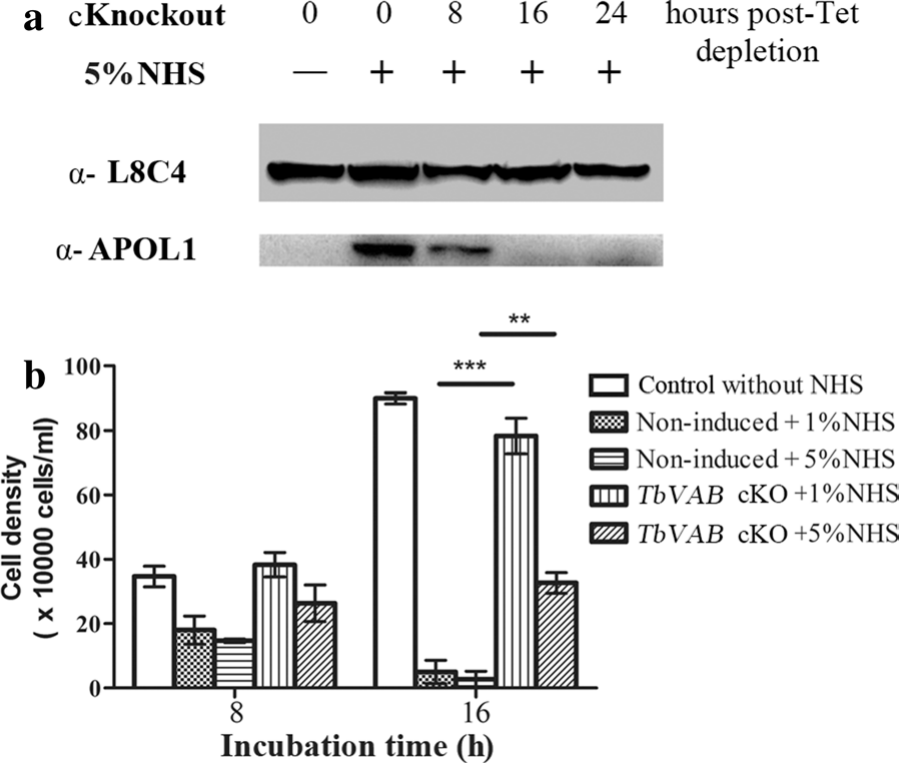
Depletion of *TbVAB* renders trypanosome cells less sensitive to trypanolysis by normal human serum. **a** Western blot analysis detecting endocytosed TLF shows relative uptake in non-induced and *TbVAB* depleted cells after incubation with 5% NHS at indicated time points. L8C4 was used as a constitutive control. **b** Trypanosome cell density was measured after incubation with normal human serum for 8h or 16h. Induced *TbVAB* depletion cells and non-induced cells were subjected to incubation with NHS. Error bars represent the standard deviation of biological triplicates. The unpaired *t*-test indicated significant differences (**P < 0.01, ***P < 0.001) between non-induced and cKO induced cells

As the endocytosis-related function of V-ATPase depends on the specific receptor/cargo, this raises the question of whether it also influences uptake of trypanosome lytic factor (TLF). Considering that both TLF-1 and -2 contain APOL1 and haptoglobin-related protein, TLF uptake could be assessed by immunoblotting with anti-APOL1 antibodies. The uptake of TLF in *TbVAB* cKO 24 hpd cells was at a distinctively low level (about 30% of the 0 hpd cells’ level at 8 hpd and later undetectable at 16 or 24 hpd) (Fig. 6a). Consistent with the low uptake of TLF, we found that *TbVAB* cKO cells, 8 hpd before 8 or 16 h NHS incubation, exhibited a high resistance to either 1% or 5% NHS treatment (Fig. 6b).

## Discussion

In this work we report, for the first time, the multiple localization of V-ATPase in the bloodstream forms of *T. brucei*. This feature suggests potential involvement of the complex in the function of co-localized organelles. Previous studies showed that V-ATPase mainly contributes to H^+^ gradient generation in acidocalcisomes and acidification in other vesicles as well as pH_i_ regulation and Ca^2+^ homeostasis in *T. brucei* [5, 22, 23]. It has also been reported that V-ATPase contributes to the action of other membrane pumps/channels, such as Ca^2+^/H^+^ exchangers, Na^+^/H^+^ exchangers, Ca^2+^ transporters, Zn^2+^ transporters, pyruvate transporters and other H^+^-ATPase(s) in *T. brucei* [19, 22, 35]. Therefore, the multiple functions of V-ATPase in *T. brucei* may reflect its position as a vital node in the ion distribution network. In our hands, by eliminating potential nonspecific interference by chemical compound inhibitors, we provided direct evidence of vacuolar and cytoplasmic alkalinization in *TbVAB* depleted BSF of *T. brucei*. *T. brucei* maintains a constant pH_i_ over a narrow range of extracellular pH [36], which we measured as a pH_i_ of 7.016 ± 0.013. This value is in a good agreement with some studies [37–39], but lower than others [22, 40]. The discrepancy in pH_i_ values might be due to different choices of pH reporters or probes. We suspect that these abnormalities in the pH homeostasis in the cytoplasm of V-ATPase ablated BSF of *T. brucei* might be due to abnormal proton efflux activity occurring as a result of the coordinated effects of V-ATPase and membrane pumps.

The localization of *T. brucei* V-ATPase in lysosomes and, probably endosomes, suggests the potential involvement of V-ATPase in endocytosis linked protein trafficking. We showed that endocytosis in *TbVAB* depleted BSF was linked to a significant decrease in lectin and TLF uptake. Hence, we consider that V-ATPase may possibly act as a specific endocytosis regulator in the BSF *T. brucei*. In contrast to lectin and TLF uptake, endocytosed transferrin was elevated in *TbVAB* depleted cells and this was consistent with another study using V-ATPase subunit F knockdown in *T. brucei* [5]. In fact, selective endocytosis, induced by endocytic protein silencing in *T. brucei*, is a common phenomenon such as in *ATG24* silencing which impairs the uptake of transferrin but not lectin [41]. Ablation of *TbRab11* has less of an effect on trafficking of GPI anchored proteins but strongly influenced recycling of transmembrane proteins [42]. Taken together these data suggest that, in the BSF *T. brucei*, V-ATPase may be a key component in endocytosis and determine specific cargo/receptor-dependent interactions.

The endocytic process and endosomal sorting regulatory mechanisms have been well studied in *T. brucei* [43, 44]. Other studies, such as effects of silencing endocytosis regulators on cell morphology, may help to understand the possible mechanisms that link V-ATPase to the endocytic process. By possessing a subpellicular microtubule cytoskeleton, *T. brucei* restricts endocytosis and exocytosis to the flagellar pocket (FP) [45]. Endocytic mutant phenotypes such as FP enlargement can be observed in examples like the ‘BigEye’ trypanosome, caused by ablation of clathrin [46]. Interestingly, however, FP enlargement was only very occasionally observed in *TbVAB* depleted BSF *T. brucei* suggesting that V-ATPase might not affect clathrin function. Also endocytosed lectin dramatically decreased while the flagellar pocket lectin was retained in *TbVAB* depletion suggesting suppression of pNAL-glycoprotein, the lectin receptor involved in endocystosis. We also suggest that V-ATPase activity might play a role in actin-dependent endocytosis as decreased lectin uptake was also found in actin ablated BSF of *T. brucei* [47]. Indeed, such links between actin and V-ATPase have been revealed in insect and mammalian cells [48, 49]. However, as endocytosed lectin decreased and had a distinctly non-lysosomal localization in Rab7 ablated BSF cells [50], the role of V-ATPase in regulating endocytosis probably differs from the Rab7 associated mechanism. Despite the diversity of endocytic defects, both actin and Rab7 ablated trypanosomes show significant resistance to NHS lysis [47, 50] and this is in agreement with our findings for *TbVAB*.

The trypanolysis caused by NHS is a serial process of initial TLF uptake in the flagellar pocket, progressive acidification in endosomal vesicles and formation of pores in the membrane. Theoretically, any barrier in the process described above will prevent the trypanolysis of trypanosomes. The SRA protein has evolved as a barrier to pore formation by APOL1 in *T. brucei rhodesiense* conferring human serum resistance [3]. Endosomal alkalinization, such as chloroquine treatment, can block trypanolysis by TLF in *T. brucei* [51]. Also the rate of uptake of TLF in *T. brucei* directly impacts NHS sensitivity in surface receptor TbHpHbR defective cells [52] and *T. brucei* 427-800 cells [51]. In the case of V-ATPase knockdown trypanosomes, previous studies have revealed that raised endosomal pH caused by the knockdown may contribute to the reduced sensitivity to NHS-mediated trypanolysis [5]. Here, we have added further important evidence that the defects in uptake of TLF, in *TbVAB* depleted BSF of *T. brucei*, may also bolster the resistance to NHS lysis, as it occurs prior to endosomal alkalinization. Considering the relationship between pH_i_/endosomal acidity and TLF uptake, treatment with chloroquine alkalinizes endosomes but causes accumulation of intracellular TLF [51, 53], suggesting that the acidic pH in the endosomes is not correlated with the uptake of TLF. Additionally, endocytosed APOL1 could lyse trypanosomes by utilizing a novel pathway of mitochondrial membrane permeabilization [54]. Therefore, a single blockage by endosomal alkalinization may not be sufficient for establishing complete TLF resistance, which is suggested by our identification of a relationship between endocytosis and V-ATPase in the BSF of *T. brucei*.

Differences in endocytic activity using different cargoes after ablation of V-ATPase subunits may be due to corresponding receptor(s) that mediate the endocytosis pathway in *T. brucei*. Lectin binds efficiently to membrane pNAL-glycoproteins [55] whilst the majority of TLF-1 binds to the GPI-anchored surface receptor TbHpHbR [56]. In the case of TLF-2, it has been proposed that TLF-2 enters trypanosomes by binding to the VSG coat and utilizing high-mannose containing residues in the binding interaction [57]. The lipoprotein receptor pathway and fluid-phase pathway may also make a minor contribution to the uptake of TLF-1/2 [58]. Our results cannot be used to distinguish the specific proportion of endocytosed TLF-1 or -2 in endocytosis-defective V-ATPase depleted trypanosomes.

## Conclusions

This study demonstrates, for the first time to our knowledge, that the V-ATPase subunit B (TbVAB) is localized in acidocalcisomes, lysosomes and probably endosomes of the bloodstream form *T. brucei*. V-ATPase is a critical proton pump complex in the endomembrane system of *T. brucei* bloodstream forms, which functions in the acidification of organelles, pH_i_ homeostasis, and endocytic regulation. *TbVAB* depleted bloodstream forms of *T. brucei* increased their endocytic uptake of transferrin and decreased their uptake of lectin and TLF, resulting in a reduced sensitivity to trypanolysis by normal human serum. Suppression of V-ATPase activity could potentially benefit the evolution of trypanosomes by improving trypanolytic tolerance. This study provides a deeper interpretation of the roles of V-ATPase in endocytic activity in *T. brucei* BSF physiology.

## Supplementary information

**Additional file 1: Table S1.** Primers used for PCR in this study.

**Additional file 2: Figure S1.** Immunoblotting of BSF and PCF of *Trypanosoma brucei* probed with rabbit polyclonal antibodies against TbVAB. TbVAB was detected by resolving whole cell lysates samples of bloodstream forms (SmOx B427) and procyclic forms (SmOx PAnTat 1.1) of *T. brucei* on 10% Tris-SDS-PAGE gels followed by western blotting probed with rabbit anti-TbVAB polyclonal antibodies. TbVAB::YFP and TbVAB were detected by resolving whole cell lysates sample of *in situ* monoallelic TbVAB::YFP labeled bloodstream forms of *T. brucei* with rabbit anti-TbVAB polyclonal antibodies. M, molecular mass markers.

**Additional file 3: Figure S2.** Validation of the *TbVAB* knockout and homologous recombination strategy for the deletion of *TbVAB*. The gel shows the PCR assays. PCR using primers targeting UTR flanking sequences and gene internal sequences demonstrated that the original alleles were disrupted (line 3). For single *TbVAB* allele replacement by homologous recombination, the *TbVAB* gene locus was initially disrupted by integration of a linear fragment containing *TbVAB* UTR and a hygromycin gene. After obtaining a single allele replaced population, an additional tetracycline inducible overexpression *TbVAB* fragment was integrated into the genome and followed by the integration of a linear fragment containing *TbVAB* UTR and a neomycin gene. *PARP*, procyclic acidic repetitive protein promoter. *T7RNAP*, bacteriophage T7 RNA polymerase. *ALD*, aldolase derived UTRs. *ACT*, actin derived UTRs. *Tetr*, Tet repressor. *HYG*, hygromycin phosphotransferase gene. *NEO*, neomycin phosphotransferase gene.

## Data Availability

All relevant information has been included in the manuscript and its additional files. Data analyzed in this study are available from the corresponding author upon reasonable request.

## Abbreviations

AO: acridine orange
APOL1: apolipoprotein L1
BCECF-AM: 2′, 7′-bis (2-carboxyethyl)-5, 6-carboxyfluorescein acetoxymethyl ester
BSF: bloodstream forms
cKO: conditional knockout
hpd: hours post-tetracycline depletion
KD: RNAi knockdown
NHS: normal human serum
PCF: procyclic form
pH_i_: intracellular pH
TLF: trypanosome lytic factors
TbVAA: V-ATPase subunit A
TbVAB: V-ATPase subunit B
V-ATPase: vacuolar H^+^-ATPase
