# Supplementary figures and images for: Vacuolar ATPase depletion contributes to dysregulation of endocytosis in bloodstream forms of Trypanosoma brucei
Source: Parasit Vectors. 2020 Apr 25;13:214. doi: 10.1186/s13071-020-04068-4 (PMC7183646; doi:10.1186/s13071-020-04068-4)

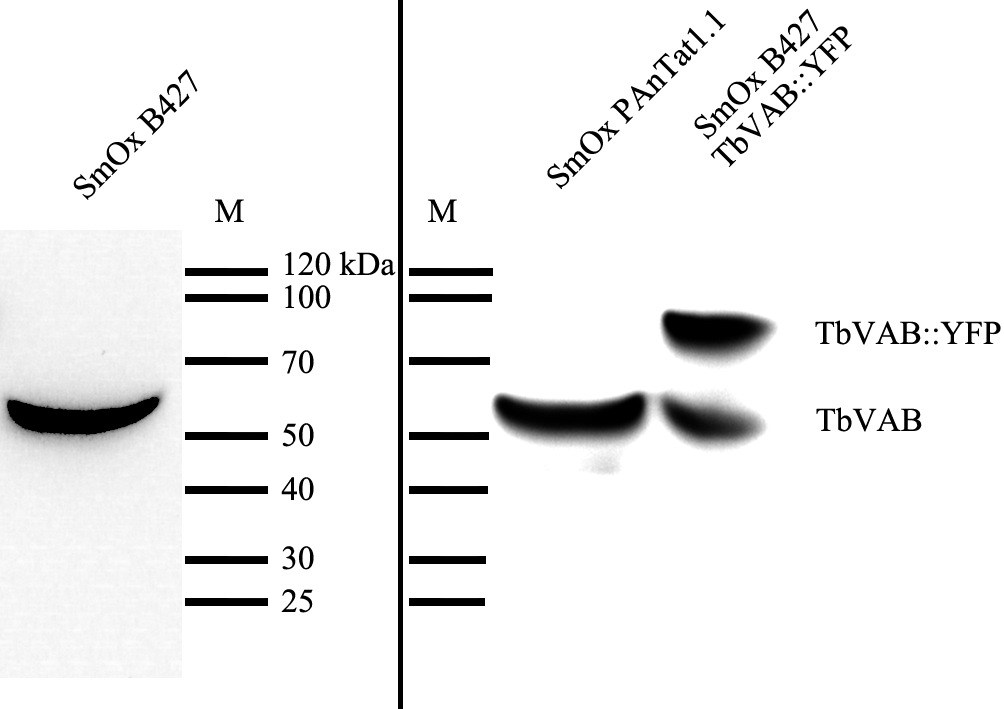

Supplement: Supplementary file 2 — Additional file 2: Figure S1. Immunoblotting of BSF and PCF of Trypanosoma brucei probed with rabbit polyclonal antibodies against TbVAB. TbVAB was detected by resolving whole cell lysates samples of bloodstream forms (SmOx B427) and procyclic forms (SmOx PAnTat 1.1) of T. brucei on 10% Tris-SDS-PAGE gels followed by western blotting probed with rabbit anti-TbVAB polyclonal antibodies. TbVAB::YFP and TbVAB were detected by resolving whole cell lysates sample of in situ monoallelic TbVAB::YFP labeled bloodstream forms of T. brucei with rabbit anti-TbVAB polyclonal antibodies. M, molecular mass markers. [file 13071_2020_4068_MOESM2_ESM.tif]

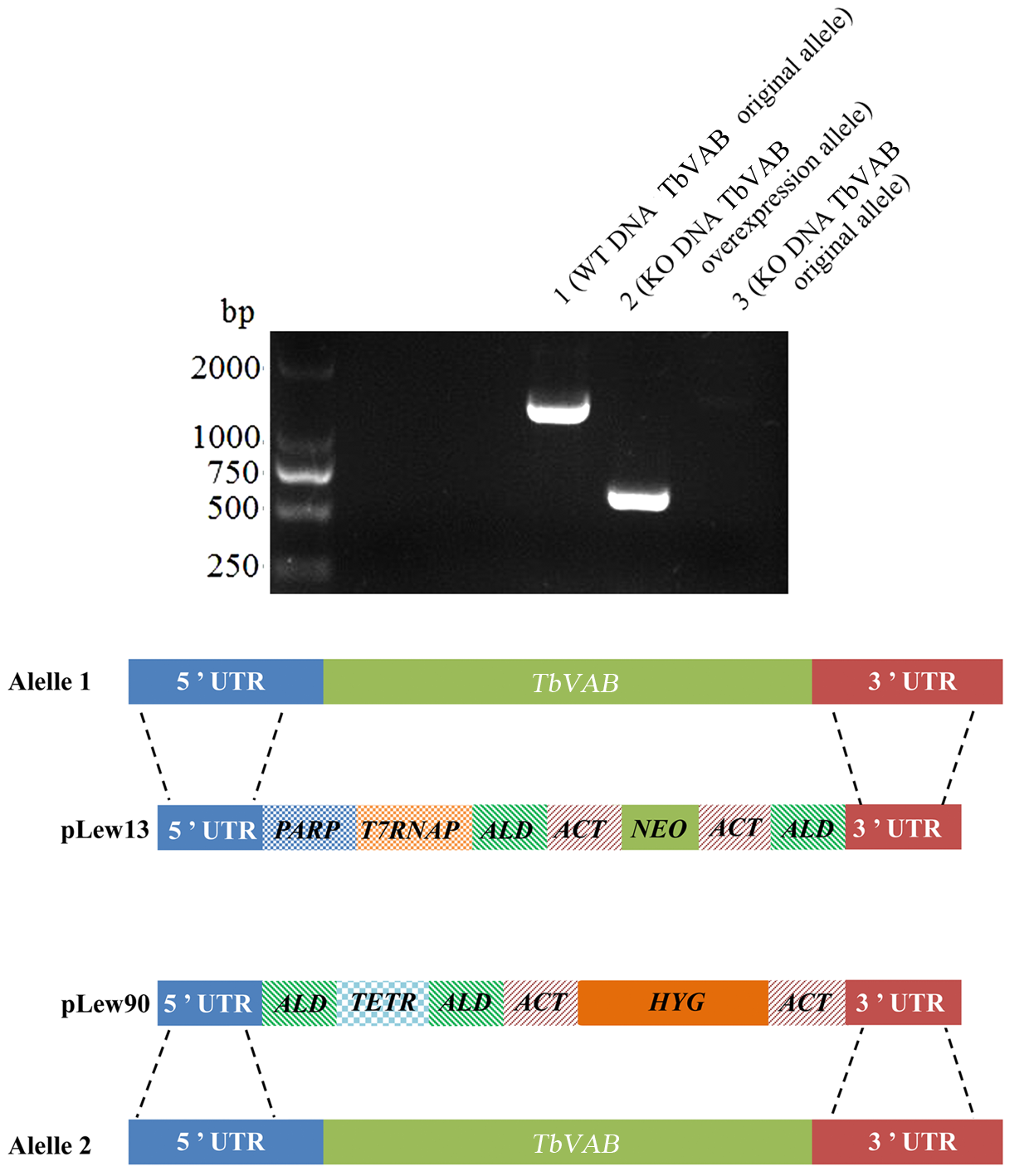

Supplement: Supplementary file 3 — Additional file 3: Figure S2. Validation of the TbVAB knockout and homologous recombination strategy for the deletion of TbVAB. The gel shows the PCR assays. PCR using primers targeting UTR flanking sequences and gene internal sequences demonstrated that the original alleles were disrupted (line 3). For single TbVAB allele replacement by homologous recombination, the TbVAB gene locus was initially disrupted by integration of a linear fragment containing TbVAB UTR and a hygromycin gene. After obtaining a single allele replaced population, an additional tetracycline inducible overexpression TbVAB fragment was integrated into the genome and followed by the integration of a linear fragment containing TbVAB UTR and a neomycin gene. PARP, procyclic acidic repetitive protein promoter. T7RNAP, bacteriophage T7 RNA polymerase. ALD, aldolase derived UTRs. ACT, actin derived UTRs. Tetr, Tet repressor. HYG, hygromycin phosphotransferase gene. NEO, neomycin phosphotransferase gene. [file 13071_2020_4068_MOESM3_ESM.tif]
